# Long Dissociation of Bictegravir from HIV-1 Integrase-DNA Complexes

**DOI:** 10.1128/AAC.02406-20

**Published:** 2021-04-19

**Authors:** Kirsten L. White, Nathan Osman, Ernesto Cuadra-Foy, Bluma G. Brenner, Devleena Shivakumar, Federico Campigotto, Manuel Tsiang, Philip A. Morganelli, Nikolai Novikov, Scott E. Lazerwith, Haolun Jin, Anita Niedziela-Majka

**Affiliations:** a Gilead Sciences, Inc., Foster City, California, USA; b McGill University AIDS Centre, Lady Davis Institute for Medical Research, Jewish General Hospital and Department of Microbiology and Immunology, McGill University, Montreal, Quebec, Canada

**Keywords:** HIV, bictegravir, dolutegravir, drug resistance, integrase, integrase inhibitors

## Abstract

The HIV integrase (IN) strand transfer inhibitor (INSTI) bictegravir (BIC) has a long dissociation half-life (*t*_1/2_) from wild-type IN-DNA complexes: BIC 163 h > dolutegravir (DTG) 96 h > raltegravir (RAL) 10 h > elvitegravir (EVG) 3.3 h. In cells, BIC had more durable antiviral activity against wild-type HIV after drug washout than RAL or EVG. BIC also had a longer *t*_1/2_ and maintained longer antiviral activity after drug washout than DTG with the clinically relevant resistance IN mutant G140S+Q148H. Structural analyses indicate that BIC makes more contacts with the IN-DNA complex than DTG mainly via its bicyclic ring system, which may contribute to more prolonged residence time and resilience against many resistance mutations.

## Abstract (precis)

The HIV integrase (IN) strand transfer inhibitor (INSTI) bictegravir (BIC) has a long dissociation half-life (*t*_1/2_) from wild-type IN-DNA complexes: BIC 163 h > dolutegravir (DTG) 96 h > raltegravir (RAL) 10 h > elvitegravir (EVG) 3.3 h. In cells, BIC had more durable antiviral activity against wild-type HIV after drug washout than RAL or EVG.

## INTRODUCTION

HIV integrase (IN) is essential for viral replication, and the IN strand transfer inhibitors (INSTIs) bictegravir (BIC), dolutegravir (DTG), elvitegravir (EVG), and raltegravir (RAL) are potent antiretroviral drugs (1). All currently approved INSTIs share a pharmacophore consisting of a metal binding scaffold that effectively chelates the two active-site catalytic Mg^2+^ ions and prevents terminal viral nucleotide 3′-OH nucleophilic attack on the host DNA, thereby blocking integration (Table 1). These INSTIs show potent inhibition of HIV-1 replication in cells; however, there are distinct differences in their chemical structures, interaction with HIV-1 IN-DNA complexes, resistance profiles, and dissociation rates from complexes of IN bound to double-stranded DNA (IN-DNA complexes) (2–9). The apparent dissociation rate of DTG from IN-DNA complexes was previously shown to be longer than those of RAL and EVG and was predicted to correlate with potent antiretroviral activity and a higher genetic barrier to resistance, but direct comparison to BIC has not been investigated (2, 3). Here, the dissociation half-life (*t*_1/2_) of BIC and other INSTIs were determined from wild-type (WT) and the clinically relevant G140S+Q148H drug-resistant mutant IN bound to IN-DNA complexes *in vitro*. In cells, INSTI dissociation was measured as viral replication after drug washout. Molecular models of BIC and DTG bound to IN-DNA complexes based on recent cryogenic electron microscopy (cryo-EM) structures provide structural insights into optimal binding geometry (2).

**TABLE 1:** Structure and antiviral activity of INSTIs in the MT-2 cell line

INSTI^*a*^	Structure	Antiviral activity against HIV-1, EC_50_ (nM)^*b*^ (fold change)	
		WT	G140S+Q148H
BIC	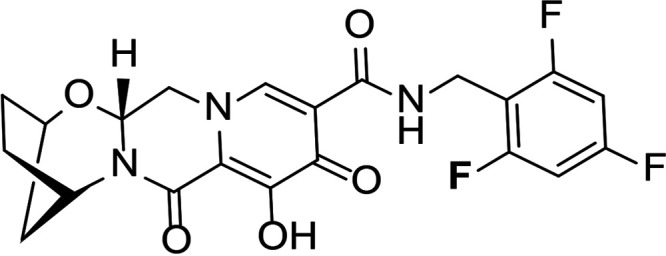	1.5 ± 0.2	3.1 ± 0.8 (2.1)
DTG	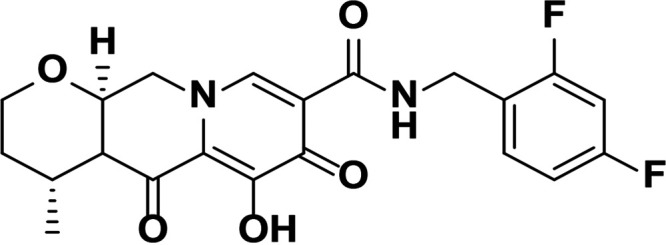	1.5 ± 0.2	6.5 ± 1.2 (4.3)
RAL	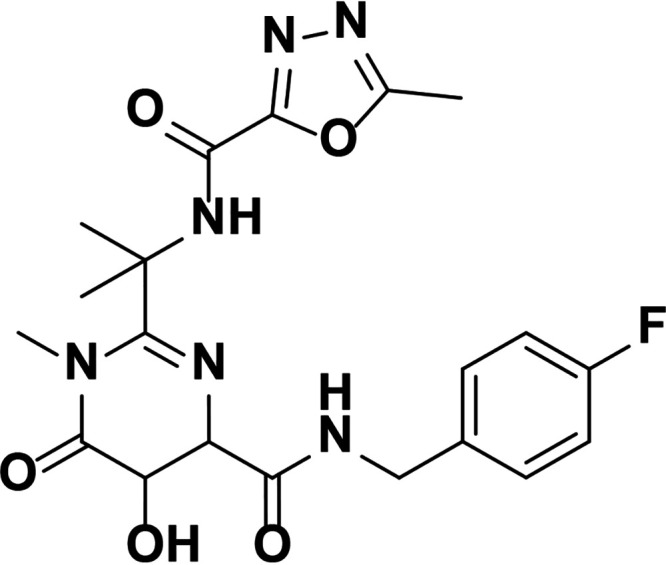	9.4 ± 1.4	2,461 ± 323 (262)
EVG	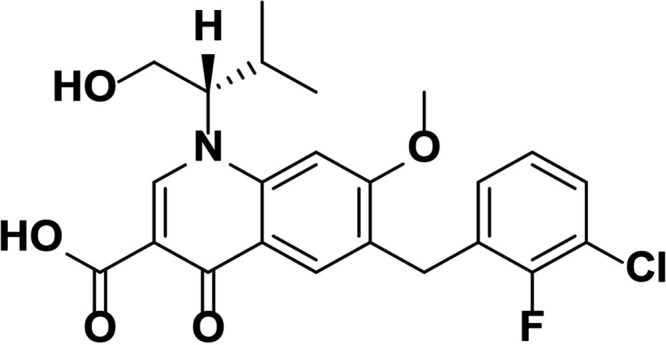	2.1 ± 0.9	868 ± 138 (413)
Compound 1	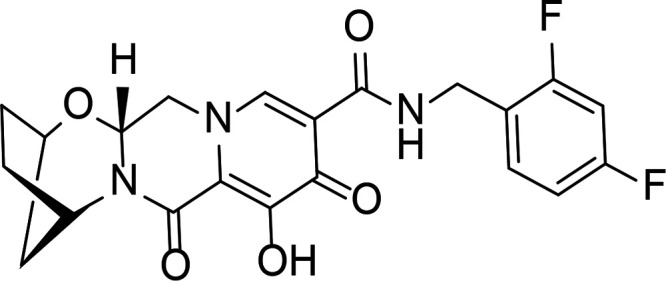	1.4 ± 0.5	4.5 ± 1.8 (3.2)
Compound 2	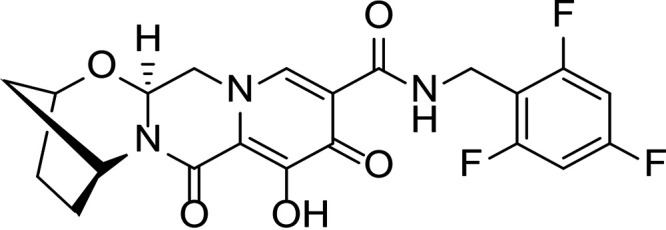	1.5 ± 0.5	11.1 ± 0.5 (7.4)

a All compounds were synthesized at Gilead Sciences, Inc.

b EC_50_, 50% effective concentration.

INSTI potency against WT HIV-1 has been well studied and, for the compounds described here, falls into the single-digit nanomole range (Table 1). BIC and DTG retain antiviral activity against many viral strains with INSTI resistance-associated mutations but show reduced activity against some complex mutants (4–9). The clinically relevant G140S+Q148H confers high-level resistance to RAL and EVG, 4.3-fold reduced susceptibility to DTG, and 2.1-fold reduced susceptibility to BIC. To further understand the role of the benzyl tail and bicyclic ring system of BIC, we studied two related analogs: compound 1 differs from BIC in that it contains a 2,4-difluorobenzyl group compared to a 2,4,6-trifluorobenzyl moiety in BIC, and compound 2 is the enantiomer of BIC (the bicyclic ring system is flipped in the opposite orientation). Compounds 1 and 2 showed more resistance to G140S+Q148H than BIC, and the orientation of the bicyclic ring system (compound 2 versus BIC) had a larger impact on mutant activity than the differences in the benzyl tail (compound 1 versus BIC).

To understand the reduced susceptibility of INSTIs to WT and G140S+Q148H IN, *t*_1/2_ values were determined by a scintillation proximity assay as described in Hightower et al. (3). Once the maximal association of [^3^H]-INSTIs to IN-DNA complexes was achieved, INSTI dissociation was initiated by adding excess unlabeled INSTI, and the signal was measured for several days to weeks (Table 2). Data sets were analyzed using a single exponential decay equation as previously published for INSTIs (3). For WT IN, BIC had a substantially longer *t*_1/2_ (163 ± 31 h) than DTG (96 ± 29 h), RAL (10 ± 2 h), and EVG (3.3 ± 0.9 h). The DTG, RAL, and EVG *t*_1/2_ values were comparable to those previously published using this methodology (3). Both compound 1 and compound 2 had *t*_1/2_ values that were intermediate to BIC and DTG. This suggests that both presence and orientation of the bicyclic ring system and the trifluorobenzyl tail are important for longer WT *t*_1/2_s.

**TABLE 2:** Dissociation half-lives and off rates of INSTIs from HIV-1 IN-DNA complexes

INSTI	WT^*a*^			G140S+Q148H^*a*^		
	Apparent *t*_1/2_ (h)^*b*^	*k*_off_ (s^−1^) (×10^−6^)^*b*^	*P* value^*c*^	Apparent *t*_1/2_ (h)^*b*^ (fold change vs WT)	*k*_off_ (s^−1^) (×10^−6^)^*b*^ (fold change vs WT)	*P* value^*c*^
BIC	163 ± 31	1.2 ± 0.3	5.7 ± 0.4 (29)	34 ± 2 (0.04)
DTG	96 ± 29 [71]	2.2 ± 0.7 [2.7 ± 0.4]	0.0019	1.9 ± 0.2 (51) [3.3]	100 ± 9 (0.02) [58 ± 8]	0.333
RAL	10 ± 2 [8.8]	21 ± 6 [22 ± 2]	0.0003	ND [0.2]	ND [1,130]	ND
EVG	3.3 ± 0.9 [2.7]	62 ± 16 [71 ± 4]	<0.0001	ND [ND]	ND [ND]	ND
Compound 1	155 ± 25	1.3 ± 0.2	0.594	4.8 ± 0.2 (32)	40 ± 2 (0.03)	0.2
Compound 2	152 ± 35	1.3 ± 0.3	0.776	1.7 ± 0.2 (89)	114 ± 12 (0.01)	0.2

a Values in square brackets are from reference 3. ND, not determined. The scintillation proximity assays for determination of HIV-1 INSTI and IN-DNA complex *t*_1/2_ values were conducted according to the protocol defined in Hightower et al. (3) but measured using the HIDEX Sense microplate reader (model 425-312, version 0.5.5.0; HIDEX, Tirku, Finland) and were maintained at 37°C. Recombinant WT and G140S+Q148H mutant HIV-1 IN enzymes containing an N-terminal 6-histidine tag (6His-IN) were purified as described in Jones et al. (22). INSTIs were tritiated by ViTrax (Placentia, CA) and had specific activities of 16.8 to 21.3 Ci/mmol. Streptavidin-coated scintillation proximity assay imaging beads (PerkinElmer, Boston, MA) were used, and oligonucleotides were obtained from Trilink (San Diego, CA), as described in Hightower et al. (3). The single exponential decay analysis was done as in Hightower et al., with the exception that we set background decay to 5%. The apparent dissociation rate constant was determined by curve fitting the competition binding phase after subtraction of 5% background to the 2-parameter single exponential decay equation: *y* = *M*(*e*^−*k*off·*t*^), where *M* is the relative binding measured at the first time point of a dissociation phase and *k*_off_ is an apparent dissociation rate constant. The half-life, *t*_1/2_, of the complex of INSTI bound to IN-DNA was calculated according to the equation *t*_1/2_ = (ln 2)/*k*_off_ and is a time needed for half of the complexes to dissociate to their individual components.

b Average ± standard deviation of 50% effective dose from 5 to 9 experiments for BIC, DTG, RAL, and EVG and 2 experiments for compounds 1 and 2.

c BIC versus other INSTI comparisons of log_10_(*k*_off_) are based on the exact Wilcoxon rank sum test.

The dissociation of INSTIs from G140S+Q148H mutant IN-DNA complexes was also studied and showed that BIC had a *t*_1/2_ of 5.7 ± 0.4 h compared to 1.9 ± 0.2 h for DTG. There was high-level resistance to RAL and EVG with G140S+Q148H mutants, resulting in an insufficient level of binding of these INSTIs to IN-DNA complexes for determination of dissociation kinetics. The *t*_1/2_ values of compounds 1 and 2 from G140S+Q148H IN were also different from those of BIC and DTG. Compound 1 retained a longer *t*_1/2_ for this mutant than DTG, whereas compound 2 had a shorter *t*_1/2_, suggesting that both the presence and the specific orientation of the bicyclic ring system in BIC are more crucial than the benzyl tail modifications.

**FIG 1:**
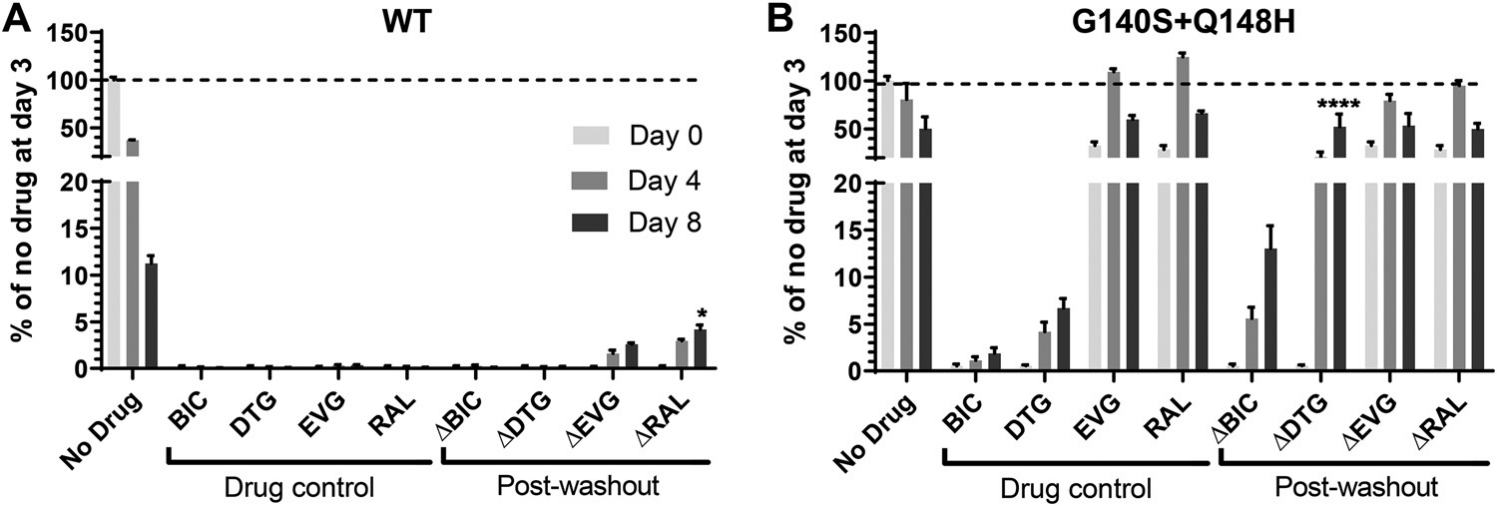
Viral replication of WT (A) and G140S+Q148H (B) variants after treatment with DTG, BIC, EVG, and RAL following drug washout (Δ) starting at washout day 0 (3 days postinfection). Drug levels corresponded to 20 times the 90% inhibitory concentration in this system (38 nM for BIC, 41.7 nM for DTG, 93.75 nM for RAL, and 24 nM for EVG). Replication was assessed by measuring RT activity in culture supernatant without and after drug washout. Bar graphs, mean ± SEM. Statistical significance of drug washout with drug control conditions were assessed by adjusted *P* value with Tukey’s test: *, <0.05; ***, <0.001; and ****, <0.0001. For the washout experiments, BIC and EVG were synthesized at Gilead Sciences, Inc., DTG was purchased from Toronto Research Chemicals (ON, Canada), and RAL was provided by Merck, Inc.

To probe the impact of the longer *t*_1/2_ of BIC compared with other INSTIs in cells where all cellular intasome components are present, we used HIV-1 strain NL4.3 containing WT or G140S+Q148H to infect MT-2 cells and treated with BIC or other INSTIs. We then washed away the inhibitors 3 days postinfection and quantified the reverse transcriptase activity in the culture supernatant to evaluate viral growth as described on washout days 0, 4, and 8 (10, 11). For BIC and DTG, the WT viral replication remained inhibited through at least 8 days after washout (Fig. 1A). EVG and RAL allowed for detectable viral replication 4 days after washout. These results are consistent with BIC and DTG having longer *t*_1/2_s from the IN-DNA complex than EVG and RAL. Longer experiments would be required to further differentiate BIC and DTG in washout experiments using WT virus.

The G140S+Q148H mutations cause varied degrees of resistance to INSTIs. In previous washout experiments, G140S+Q148H-containing virus was able to resume replication and integration after washout of RAL or EVG (10). Even though very low levels of replication were observed under constant BIC pressure, BIC was able to maintain >80% of suppression of the G140S+Q148H virus after 8 days of its washout (Fig. 1B). In contrast, there was low replication under constant DTG pressure and significant viral replication 4 and 8 days after DTG washout. These data obtained in the more complex cell-based assay support the biochemical *t*_1/2_ results. BIC maintained antiviral activity after washout for several days against WT and mutant HIV, which provides further evidence that long *t*_1/2_s may prevent viral rebound and emergent resistance *in vivo* after missing doses of drug. These findings are consistent with the resistance profiles reported in Table 1.

**FIG 2:**
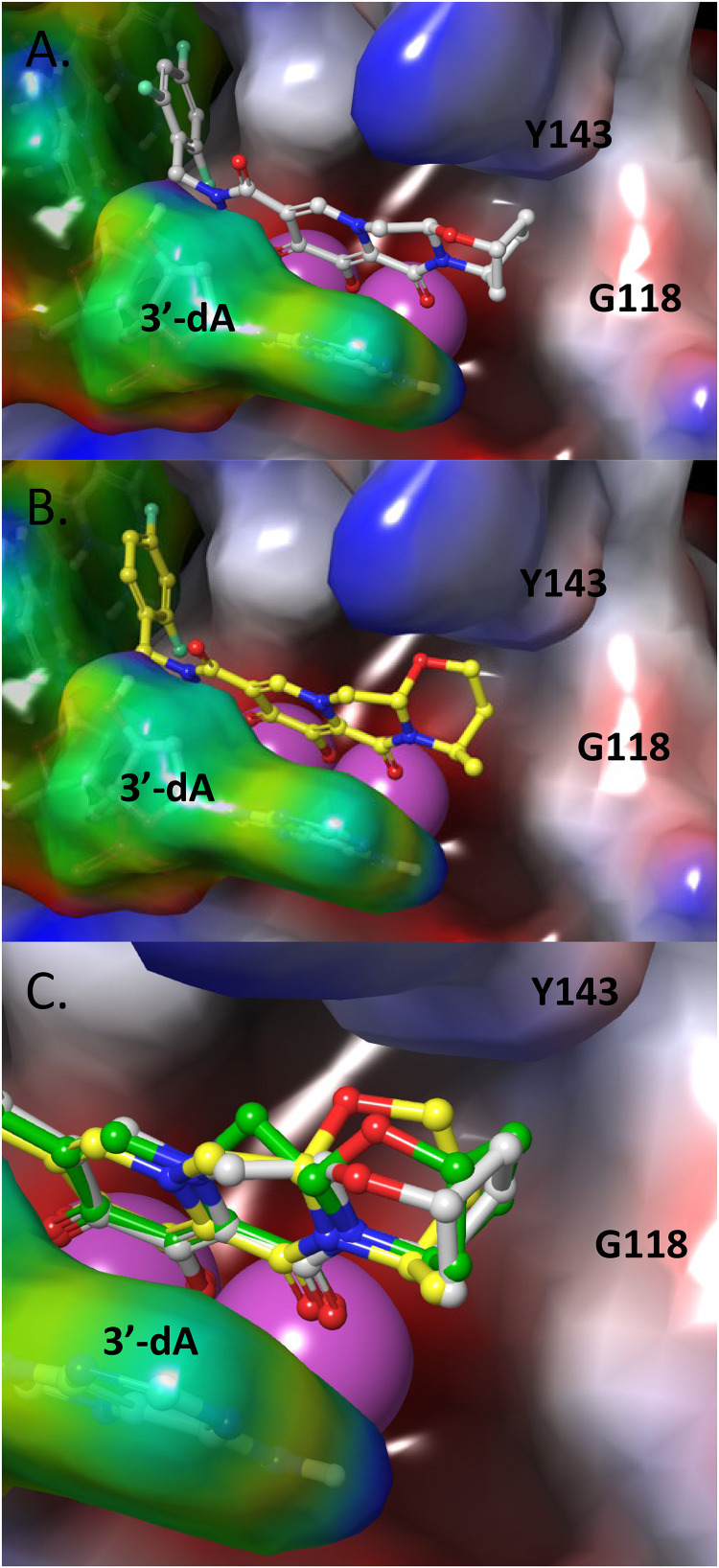
Molecular models of BIC (A, gray), DTG (B, yellow), and overlap (C) bound to the HIV-1 IN active site. HIV-1 IN and vDNA are shown in blue-red-white and rainbow electrostatic surface representation, respectively. The two Mg^2+^ ions are shown in magenta spheres. The bicyclic A-ring of BIC makes van der Waals contacts with the IN β4-α2 loop (G118 shown for reference) and 3′-dA of the vDNA, filling up this region of the binding pocket efficiently and acting as additional anchoring points. DTG with its monocyclic ring and flipped stereochemistry makes partial contact with this region. Compound 2 (C, green) is a stereoisomer of BIC (gray) where the bicyclic ring points away from the 3′-dA vDNA. The optimal contacts of BIC with IN β4-α2 loop and vDNA may be major contributors to anchoring the inhibitor in the pocket. Methodologies for the models are as follows. The INSTIs were docked to a homology model of WT and G140S+Q148H mutant HIV-1 IN based on cryo-EM structures (SIVrcm IN) (2) using Prime in Schrodinger Suite 2019-2 (Schrödinger, LLC, New York, NY) and a knowledge-based approach (23). The sequence alignment between SIVrcm IN (template) and HIV-1 IN (query) was optimized using ClustalW (24), yielding a sequence identity of 73%, a sequence similarity of 83%. The residues that were similar between the two sequences were retained while building the model. The side chains of the residues that were not part of the template were iteratively sampled using a coarse library of rotamers derived from known PDB structures until no clashes remained. The coordinates of all atoms not derived directly from the template itself were then minimized, producing the final refined model. BIC, DTG, and compound 2 were prepared with LigPrep with the metal binding states option using Schrodinger Suite 2019 (Schrödinger). INSTIs were docked using Glide XP docking protocol with expanded sampling method (25). Solvent molecules that coordinate to active site Mg^2+^ ions were kept in place during docking. Top three docked poses for both the INSTIs were saved as an output.

To understand the longer *t*_1/2_ of BIC from HIV-1 IN-DNA complexes compared with that of DTG, molecular models of the INSTIs using cryo-EM structures of BIC with simian immunodeficiency virus (SIV)rcm IN (WT and G140S+Q148H mutant) or HIV-1 IN, viral DNA (vDNA) and BIC were generated (2). Previously solved X-ray structure of prototypic foamy virus (PFV)-IN with DTG (PDB accession no. 3S3M) was also studied for predicting binding mode of DTG with HIV-1 IN. The INSTIs bind at the interface between vDNA and IN protein. We observed that several factors contribute to improved interaction between BIC and the catalytic core domain of HIV-1 IN. The trifluorobenzyl tail of BIC fills a pocket that is lined by the 3′-deoxycytosine (dC) of the vDNA and HIV-1 IN protein. This pocket would have been occupied by the terminal 3′-deoxyadenosine (dA) of vDNA in the absence of an INSTI. The favorable π-stacking interaction of the halobenzyl tail with the 3′-dC base of the vDNA helps with the potency and resistance profile. The core ring of BIC stacks with the terminal 3′-dA base of the vDNA. The metal binding pharmacophore of the core coordinates the two catalytic Mg^2+^ ions in the active site. Based on the modeled structures of BIC and DTG (Fig. 2A to C), it is evident that the bicyclic ring of BIC makes additional van der Waals contacts with the β4-α2 loop (see G118 in Fig. 2) of WT IN and 3′-dA vDNA compared to DTG, where the corresponding monocyclic ring of DTG has only partial contact with this region of HIV-1 IN. This additional interaction of the bicyclic ring of BIC with the β4-α2 loop of IN and 3′-dA vDNA may contribute to its tighter binding and explain why we observed the longer *t*_1/2_. These models also agree well with other homology models based on cryo-EM structures of HIV-1 IN with BIC (PDB accession no. 6PUW) (2, 12). A recent work using explicit solvent molecular dynamics simulations of WT and G140S+Q148H bound with BIC and a compound without a bicyclic ring system and containing 2,4-difluorobenzyl also shows the importance of additional interactions with the IN β4-α2 loop (2). A wider distribution of the atomic displacements in the case of this truncated analog compared to BIC in WT and G140S+Q148H supports the importance of this additional anchoring interaction.

Understanding the interactions between INSTIs and their targets is clinically relevant because INSTI-based regimens are the recommended choice for initial HIV therapy. The INSTIs BIC and DTG are highly potent and have high genetic barriers to resistance. Emergent resistance to three-drug BIC- and DTG-based regimens has not occurred in clinical trials but has occurred in rare cases in clinical practice (13). EVG- and RAL-based regimens, which have lower genetic barriers to resistance, select for IN mutations at codons 92, 143, 155, and 148. A long INSTI residence time may lead to higher tolerance to missed doses, but antiviral support by two nucleoside RT inhibitors of BIC- and DTG-based three-drug regimens is also important. There are some concerns around the resistance risk of potentially less forgiving two-drug INSTI-based regimens (DTG plus either lamivudine or rilpivirine) or three-drug RAL- or EVG-based regimens, which have lower resistance barriers (9, 14–17). Single-drug therapy, such as DTG monotherapy, has given rise to unacceptably high rates of virological failure with INSTI resistance (16). Likewise, cases of failure with emergent M184V and R263K were reported for DTG-plus-lamivudine dual therapy in the clinical trials GEMINI and ACTG5353 (18, 19). Clinically, the long residence times of BIC and DTG may help support their high barriers to resistance by maintaining viral inhibition during periods of low drug levels, such as when several consecutive doses are missed.

We performed enzymatic, tissue culture, and structural studies to investigate the mechanistic basis for the more favorable resistance profiles of BIC and DTG compared with EVG and RAL and to investigate the differences between BIC and DTG. Drug washout/release studies are consistent with a longer *t*_1/2_ of BIC compared to DTG. Structural studies of the INSTI interactions with integrase intasomes (2, 12, 20, 21) and our models show that BIC forms a stable complex with HIV-1 IN bound to HIV-1 long-terminal-repeat DNA. The *t*_1/2_ of BIC from HIV-1 IN-DNA complexes was longer than the *t*_1/2_ of DTG, RAL, and EVG. The long *t*_1/2_s of INSTIs with the integrase-DNA complex have been correlated with potent antiretroviral activity against WT HIV-1 integrase and a high barrier to resistance *in vitro* (3). BIC also dissociated more slowly than DTG from the resistant mutant G140S+Q148H IN-DNA complex, which is consistent with the greater *in vitro* activity of BIC than of other INSTIs against this mutant (2–9). Long *t*_1/2_ and more optimal binding-site complementarity of the INSTI into its binding site may also translate into more favorable resistance profiles when the INSTI may tolerate small perturbations in the pocket induced by amino acid mutations. The impact of a longer BIC *t*_1/2_ due to more optimal interactions with HIV-1 IN and vDNA may lead to greater tolerance of binding-site perturbations by drug resistance mutations and forgiveness of missed doses. These *in vitro* data further our understanding of the high efficacy and resistance barrier of BIC in the treatment of people living with HIV.
